# Neuroprotective Effects and Mechanism of *β*-Asarone against A*β*1–42-Induced Injury in Astrocytes

**DOI:** 10.1155/2017/8516518

**Published:** 2017-11-28

**Authors:** Yuanxiao Yang, Ling Xuan, Hongshu Chen, Shijie Dai, Liting Ji, Yuting Bao, Changyu Li

**Affiliations:** ^1^Department of Basic Medicine, Hangzhou Medical College, Hangzhou, Zhejiang 310053, China; ^2^College of Pharmacy, Zhejiang Chinese Medical University, Hangzhou, Zhejiang 310053, China; ^3^The First Affiliated Hospital of Zhejiang Chinese Medical University, Hangzhou, Zhejiang 310006, China

## Abstract

Emerging evidence suggests that activated astrocytes play important roles in AD, and *β*-asarone, a major component of* Acorus tatarinowii* Schott, was shown to be a potential therapeutic candidate for AD. While our previous study found that *β*-asarone could improve the cognitive function of rats hippocampally injected with A*β*, the effects of *β*-asarone on astrocytes remain unclear, and this study aimed to investigate these effects. A rat model of A*β*1–42 (10 *μ*g) was established, and the rats were intragastrically treated with *β*-asarone at doses of 10, 20, and 30 mg/kg or donepezil at a dose of 0.75 mg/kg. The sham and model groups were intragastrically injected with an equal volume of saline. Animals were sacrificed on the 28th day after administration of the drugs. In addition, a cellular model of A*β*1–42 (1.1 *μ*M, 6 h) was established, and cells were treated with *β*-asarone at doses of 0, 2.06, 6.17, 18.5, 55.6, and 166.7 *μ*g/mL. *β*-Asarone improved cognitive impairment, alleviated A*β* deposition and hippocampal damage, and inhibited GFAP, AQP4, IL-1*β*, and TNF-*α* expression. These results suggested that *β*-asarone could alleviate the symptoms of AD by protecting astrocytes, possibly by inhibiting TNF-*α* and IL-1*β* secretion and then downregulating AQP4 expression.

## 1. Introduction

Alzheimer's disease (AD) is the main cause of dementia and one of the great healthcare challenges of the 21st century [1]. In 2016, 44 million people were estimated to suffer from AD-related dementia worldwide [2]. AD is characterized by progressive memory loss and cognitive dysfunction, and accumulation of extracellular plaques made of amyloid-*β* (A*β*) and intracellular neurofibrillary tangles (NFT) made of tau protein are considered the main pathological features of the disease [3–6].

Nonetheless, increasing evidence suggests that pathological processes independent of A*β* plaque deposition may contribute to the initiation of AD [7, 8]. Glial cell activation and neuroinflammation are increasingly recognized as early events in the disease, even preceding A*β* plaque deposition [9, 10], potentially making glial cells a promising therapeutic target [11–14]. Astrocytes, the most numerous brain cell type, are involved in immune, physiological, and pathological reactions in the brain by secreting a large number of immune and inflammatory mediators. Studies have shown activated astrocytes in early-stage AD patients and transgenic animals [15–18], and postmortem analyses of astrocytosis in human AD brain tissues have reported activated glial fibrillary acidic protein- (GFAP-) positive astrocytes next to fibrillar A*β* plaques [15, 16]. In addition, activated astrocytes may release the proinflammatory cytokines TNF-*α*, IL-1*β*, and IL-6 and nitric oxide (NO) as well as other potentially cytotoxic molecules [19, 20], and studies have shown that astrocytes support hippocampal-dependent memory and long-term potentiation via interleukin-1 signaling [21]. A recent study found that overexpression of IL-1*β* reduced A*β*-related pathology by modulating innate immune responses or promoting nonamyloidogenic APP cleavage in mouse and cell culture models of AD, suggesting that IL-1*β* may play a beneficial role in limiting AD pathology [22, 23]. Therefore, we mainly investigated IL-1*β* as an important indicator.

The water channel protein aquaporin-4 (AQP4), mainly expressed in astrocytic endfeet, is the major aquaporin in the mammalian brain and helps maintain water homeostasis in the central nervous system [24]. Altered AQP4 expression and localization in reactive astrocytes have been observed in patients with AD and in several transgenic mouse models of AD [25, 26]. Furthermore, animal experiments demonstrated that knocking down AQP4 reduced A*β* clearance from the brain [27], and cell experiments demonstrated that AQP4 knockout reduced A*β*1–42-induced astrocyte activation and apoptosis [28]. These data suggest that the role of AQP4 in the pathophysiological process of AD should be a premise for therapeutic AD strategies.

**Figure 1:**
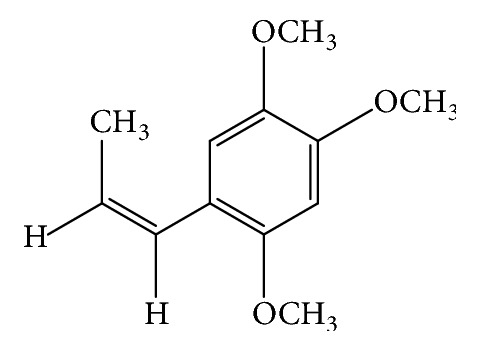
Chemical structure of *β*-asarone.

*β*-Asarone (cis-2,4,5-trimethoxy-1-allyl phenyl) is a major component of* Acorus tatarinowii* Schott, which is native to Central Asia, North America, and Eastern Europe [29]. *β*-Asarone (Figure 1) could reportedly attenuate neuronal apoptosis in rat hippocampi and might have potential as a therapeutic agent to manage cognitive impairment in a mouse model of AD [30–32]. Other authors found that *β*-asarone could prevent A*β*25–35-induced inflammatory responses and autophagy in SH-SY5Y cells [33]. In addition, *β*-asarone could protect against A*β*1–42 induced cytotoxicity in PC12 cells [34] and cerebrovascular AD rats [35]. Our previous study also found that *β*-asarone could protect against cerebral ischemia [36] and improve the cognitive functions of rats hippocampally injected with A*β*, but the effects of *β*-asarone on astrocytes remain unclear. Therefore, this study aimed to investigate the effects and associated mechanisms of *β*-asarone on astrocytes.

## 2. Materials and Methods

### 2.1. Animal and Groups

This animal study was approved by the Ethics Committee of Zhejiang Traditional Chinese Medical University. Male Sprague Dawley (SD) rats (age, 9 weeks; weight 250 ± 30 g) of SPF grade were purchased from the Chinese Academy of Sciences Shanghai Branch, Sippr-BK Laboratory Animal Center (animal production license number: SCXK (Shanghai) 2013-0016). Animals were housed at a constant room temperature (20 ± 2°C) and supplied with sterilized food and water (laboratory rearing room permit number SYXK (Zhejiang) 2013-0184). All animals were subjected to acclimatization for one week before the experiment began.

The learning and memory abilities of all the SD rats were assessed using the Morris water maze (MWM) (Smart-Mass 0800916s, Panlab, Spain) [37–39]. In total, 84 SD rats were randomly divided into the sham (12) or AD model (72) groups. Rats in the AD model group were injected with A*β*1–42 (1 *μ*g/*μ*L A*β*1–42, 5 *μ*L/each side), and those in the sham group were injected with a sterile saline solution in both sides of the CA1 area of the hippocampus. Seven days after the A*β*1–42 injection, the MWM test was performed to evaluate the learning and memory capacities of the rats. A total of 60 rats with significantly reduced learning and memory capacities were chosen and further randomly divided into the AD model group (*n* = 12), the *β*-asarone group (10 mg/kg/d, 20 mg/kg/d, and 30 mg/kg/d; *n* = 12 in each dose group), and the donepezil group (0.75 mg/kg/d; *n* = 12). Rats of the drug groups were intragastrically given *β*-asarone at doses of 10 mg/kg/d, 20 mg/kg/d, or 30 mg/kg/d or donepezil at a dose of 0.75 mg/kg/d. The sham and AD model groups were intragastrically given the same volume of saline. On the 7th, 21st, and 28th day after the operation and the 21st and 28th day after drug treatment, the MWM test was performed to evaluate the learning and memory capacities of the rats. Rats in each group were sacrificed on day 28 posttreatment. Biochemical indicator samples and organs were harvested for analysis, and the remaining samples were stored at −80°C for protein extraction.

### 2.2. Cells and Groups

Hippocampal rat RA-h astrocytes were stimulated with different concentrations of A*β*1–42 (0, 0.36, 1.1, 3.3, 10, and 30 *μ*M) for 0, 3, 6, 12, 24, and 48 h, and the optimal A*β*1–42 concentration and time were selected for subsequent analyses. The viability of RA-h cells after *β*-asarone treatment (0, 2.06, 6.17, 18.5, 55.6, 166.7, and 500 *μ*g/mL) was assessed using a fully automated high-throughput real-time system to choose the optimal concentration.

### 2.3. Injection of A*β*1–42 into the CA1 Area of the Hippocampus

SD rats were injected with 3% pentobarbital sodium (1.5 mL/kg (BW)). The head of each animal was shaved around the fontanelle region, and the head was then fixed in a brain stereopositioning instrument (Alc-H, Shanghai Alcott Biotech Co., Ltd.) and disinfected with iodophor. The injection site in the CA1 area of the hippocampus was 3.3 mm caudal to the bregma and 2.0 mm to the right and left of the midline [39]. To drill the skull with a dental drill (strong 90, Shenzhen RWD Life Science Co. Ltd.), a downward needle with a 10 mL microsyringe (Shanghai Gaoge Industry & Trade Co., Ltd.) was vertically inserted 2.8 mm from the brain surface and then retracted 1 mm to reserve a certain injection space. Next, 5.0 *μ*L of A*β*1–42 (5.0 *μ*g A*β*1–42, 120MB711V, Sigma-Aldrich Co., Ltd.) was slowly injected into the left and right sides of the brain. The injection was completed within 5 minutes, after which the needle was kept in the same position for an additional 5 minutes to ensure that the solution was fully dispersed.

### 2.4. Behavioral Tests

The MWM test [37, 39] (Smart-Mass 0800916s, Panlab, Spain), which includes place navigation and space exploration, was conducted one week before the rats were grouped and the first, second, and fourth weeks after modeling.

The MWM test was carried out in a low-light environment maintained at 24 ± 1°C in a circular tank (diameter, 90 cm; height, 50 cm) filled with water. Water was filled to 1 cm above the surface of a removable circular platform (diameter, 9.5 cm; height, 28 cm) that was located inside the pool [40].

#### 2.4.1. Place Navigation Test

**Figure 2:**
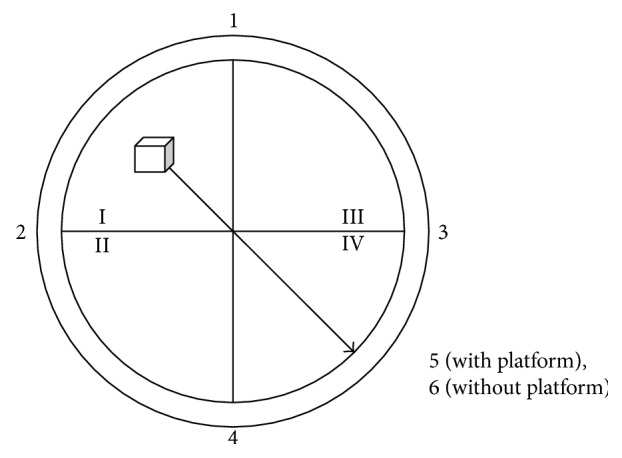
Distribution of the Morris water maze experiment entry points.

To measure the ability of rats to acquire experiences or learning, on the afternoon one day before the experiment, rats were placed into the water for 2 min to acclimate to the experimental environment. The formal experiment lasted 2.5 days, with training two times each day in the morning and afternoon (Figure 2), and the pool entry points were numbered 1–5. The rats were placed into the pool facing the pool wall; the entry point for rats was identical in the same time. The upper time limit was set to 120 s, and the time required for each group of rats to find the platform was recorded as the escape latency. If a rat did not find the platform in 120 s, it was led to the platform where it stayed for 10 s to strengthen its memory, and its escape latency was recorded as 120 s.

#### 2.4.2. Space Exploration Experiment

The platform was removed on the afternoon of the third day, and the rats were placed into the sixth entry point facing the wall of the pool. The time required for each rat to reach the platform in 120 s was recorded as the space exploration ability index.

### 2.5. Biochemical Measurements in Blood

Blood samples, acquired from the heart after 28 days of drug treatment, were centrifuged at 3000 rpm for 10 min (4°C), and all samples were stored at −80°C. The IL-1*β* and TNF-*α* hippocampal contents were measured by an enzyme-linked immunosorbent assay.

### 2.6. Brain Pathological Studies

Brain tissues were fixed in 4% paraformaldehyde and embedded in paraffin. The brain tissues were then cut into slices, and sections were stained with Congo red to observe A*β* deposition in the hippocampus. Hematoxylin and eosin (HE) and Nissl staining were also performed to evaluate brain structural injuries. Sections from each group were randomly selected and photographed under a high-powered microscope (Leica, Wetzlar, Germany).

### 2.7. Immunocytochemistry Studies

Paraffin-embedded renal sections (3–5 *μ*m) were subjected to immunohistochemical analysis. The sections were quenched with 3% H_2_O_2_ for 10 min, washed with PBS 3 times, and then incubated overnight with an anti-GFAP primary antibody (ab4648, Abcam, Cambridge, UK). Subsequently, the stained sections were incubated with peroxidase-conjugated secondary antibodies. The immunocomplexes were visualized by 3,3-diaminobenzidine (DAB) substrate, and all sections were counterstained with hematoxylin prior to mounting. Positive expression intensity was judged according to the staining area in each field, and image analysis was performed by ImagePro Plus software.

### 2.8. Cell Culture and Identification

The RA-h cell line, purchased from Shanghai SH Biotechnology Co., Ltd., was cultured in Dulbecco's Modified Eagle's Medium (DMEM) (Gibco) supplemented with 10% fetal bovine serum (FBS, Gibco) and 1% penicillin/streptomycin/glutamine (PSG, Hangzhou Genom Biopharmaceutical Technology Co., Ltd.) and incubated at 37°C in 5% CO_2_. RA-h cells were identified by immunofluorescence, which involved adding 4% paraformaldehyde (1 mL/well) for 20 min to fix the cells, adding 0.5% Triton for 10 minutes, and blocking with 5% bovine serum albumin (BSA) for 1 h. The sections were incubated first with a monoclonal mouse anti-GFAP primary antibody overnight at 4°C (ab4648, Abcam) and then with an Alexa Fluor 488 (A-11001, Life Technologies, MA, USA, dilution 1 : 1000) secondary antibody for 1 h. Nuclei were stained with 4′,6-diamidino-2-phenylindole (DAPI, R37606, Life Technologies), and coverslips with SlowFade Diamond Antifade Mountant (S36963, Life Technologies, Rockford, USA) were added. The mounted slides were observed using a microscope (DMI3000B, Leica).

### 2.9. Cell Viability Assay

The viabilities of RA-h cells after A*β*1–42 or *β*-asarone treatment were detected using a fully automated high-throughput real-time system. The real-time cell monitoring device (xCELLigence, CA92121, ACEA Biosciences Inc., Hangzhou) in this study consisted of single-use E-plates with gold microelectrode arrays covering the bottoms of the wells inserted into a real-time cellular analysis (RTCA) single-plate station located within the incubator. Fluctuations of impedance could be measured when a population of cells grew, attached, and spread on the electrode surface. All steps were performed under sterile conditions, and 100 *μ*L of a cell suspension (5 × 10^3^ cells/well) was added to E-Plate 16, which was loaded into the RTCA DP Analyzer inside the incubator for measurements.

### 2.10. Reverse Transcription Quantitative Polymerase Chain Reaction (RT-qPCR)

Total RNAs from RA-h cell samples were extracted using AxyPrep RNA Kits according to the manufacturer's manual. Then, PrimeScript RT Reagent Kits (RR037A, TaKaRa Biotechnology, Beijing) were used to synthesize cDNA, and the specific primers for the RA-h cell samples are listed in Table 1. The PCR cycling conditions were as follows: predenaturation at 95°C for 5 min, followed by 40 cycles at 94°C for 1 min, 95°C for 10 sec, 58°C for 10 sec, and 72°C for 10 sec. mRNA quantities were determined using cycle threshold (CT) values, which were calculated by the computer software. The relative mRNA expression levels were determined by the 2^−ΔΔCt^ method.

### 2.11. Western Blot Analysis

Total proteins from nephridium were extracted with RIPA lysis buffer, and equal amounts were then resolved using SDS-PAGE and transferred onto polyvinylidene difluoride (PVDF) membranes. After being blocked with 5% nonfat milk, the membranes were incubated overnight with primary antibodies, and the antibodies used included anti-GFAP (ab4648, Abcam), anti-AQP4 (ab9512, Abcam), anti-IL-1*β* (ab9722, Abcam), and anti-TNF-*α* (ab9755, Abcam). Next, the membranes were incubated with peroxidase-conjugated secondary antibodies. The optical densities of the bands were quantified using *β*-actin or GAPDH as the internal references, and immunocomplexes were visualized with an Odyssey near infrared dual color laser imaging system.

### 2.12. Statistical Analyses

Data are shown as the mean ± standard deviation (SD). GraphPad Prism 5.0 software and SPSS software (Version 17.0) were adopted for statistical analyses. Student's* t*-tests were used to compare differences between two groups, and analysis of variance (ANOVA) was used to assess statistical significance in multiple groups. *P* < 0.05 and *P* < 0.01 were considered significantly different.

## 3. Results

### 3.1. *β*-Asarone Increases Spatial Learning and Memory Abilities in Rats

**Figure 3:**
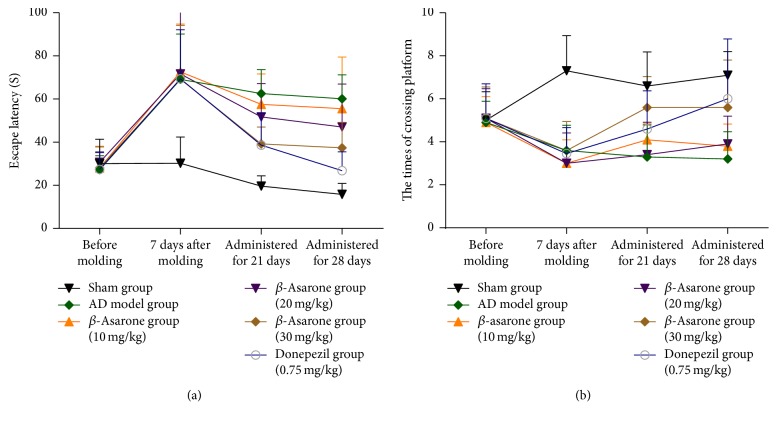
Effects of *β*-asarone on the escape latencies and times required to cross the platform in AD model rats induced by A*β*1–42.

The spatial learning and memory capacities of the rats were examined by the MWM method. As shown in Figure 3(a), one week after modeling, the escape latencies of all animals in the A*β*1–42 injection group were significantly longer than those of the sham group. After *β*-asarone treatment for 28 days, the escape latencies of rats in the *β*-asarone treatment group (30 mg/kg) were shortened compared to those of the AD model group (37.4 ± 11.82 s versus 60.1 ± 11.10 s, *P* < 0.05).

In addition, one week after modeling, the times required to cross the platform were significantly decreased in rats of the A*β*1–42 injection group compared to those of the sham group (Figure 3(b)). After *β*-asarone treatment for 28 days, the times required to cross the platform for rats in the *β*-asarone treatment group (30 mg/kg) were increased compared to those of the AD model group (5.6 ± 2.2 versus 3.2 ± 1.27, *P* < 0.01).

### 3.2. *β*-Asarone Alleviates Brain Tissue Injury in Rats

**Figure 4:**
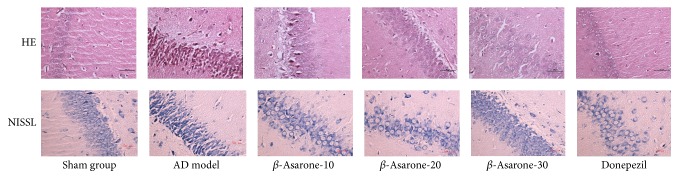
Effects of *β*-asarone on the histopathology of CA1 region neurons in AD model rats induced by A*β*1–42 (400x magnification).

As shown in Figure 4, HE staining showed that CA1 region neurons in hippocampi of the sham group were arranged in an orderly manner and had normal structures, clear nuclei, distinct nucleoli, light staining, and rich cytoplasm. By contrast, many swollen neurons with loosened structures, deep staining, pyknotic nuclei, and vacuole-like structures were observed in the AD model group one week after modeling. After *β*-asarone treatment for 28 days, all of these abnormalities were alleviated in groups receiving *β*-asarone treatment (20 mg/kg, 30 mg/kg).

In addition, the Nissl staining results suggested that CA1 region neurons in the hippocampi of sham group rats were abundant, with clear colors and kernels and weak nuclei being observed. By contrast, neuron tissues observed in the AD model group were disorganized, swollen, and deformed, with condensed and deeply stained nuclei being observed one week after modeling. After *β*-asarone treatment for 28 days, all of these abnormalities were alleviated in groups receiving *β*-asarone treatment (20 mg/kg, 30 mg/kg).

### 3.3. *β*-Asarone Alleviates Amyloid Deposition in Rats

**Figure 5:**
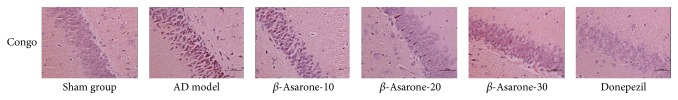
Effects of *β*-asarone on amyloid deposition in CA1 region neurons in AD model rats induced by A*β*1–42 (400x magnification).

As shown in Figure 5, no significant pathological changes were found in the sham group, while amyloid deposits were observed in CA1 region hippocampal neurons of rats in the AD model group one week after modeling. After *β*-asarone treatment for 28 days, all of these abnormalities were alleviated in groups receiving *β*-asarone treatment (20 mg/kg, 30 mg/kg).

### 3.4. *β*-Asarone Suppresses Astrocyte Activation in Rats

**Figure 6:**
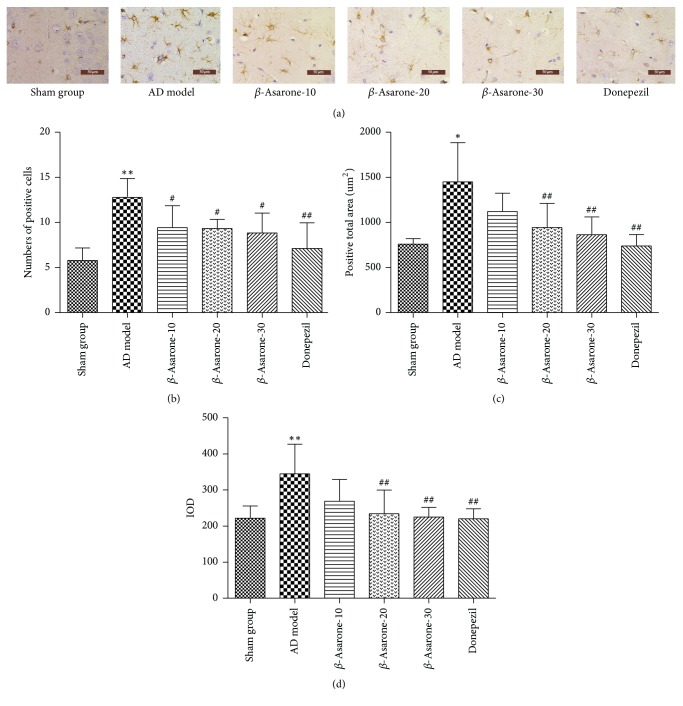
Effects of *β*-asarone on the expression of GFAP in AD model rats induced by A*β*1–42.* Note*. Compared with the sham group, ^*∗*^*P* < 0.05, ^*∗∗*^*P* < 0.01; compared with the model group, ^#^*P* < 0.05, ^##^*P* < 0.01.

Our immunohistochemistry results demonstrated that the number of positive cells (Figure 6(b)) was increased in the AD model group. Furthermore, the total area of GFAP-positive expression (Figure 6(c)) and the integral optical density (IOD) (Figure 6(d)) were significantly increased in the AD model compared to those of the sham group (1448.5 ± 438.2 versus 759.9 ± 61.0 *μ*m^2^; 344.6 ± 82.0 versus 221.9 ± 33.87) (*P* < 0.05, *P* < 0.01), and these changes were partly reversed after *β*-asarone treatment. The changes were obvious in the *β*-asarone treatment groups (20 mg/kg, 30 mg/kg) (*P* < 0.01, *P* < 0.01), proving that *β*-asarone could protect astrocytes in rat brains.

### 3.5. *β*-Asarone Decreases IL-1*β* and TNF-*α* Levels in Rat Hippocampi

**Figure 7:**
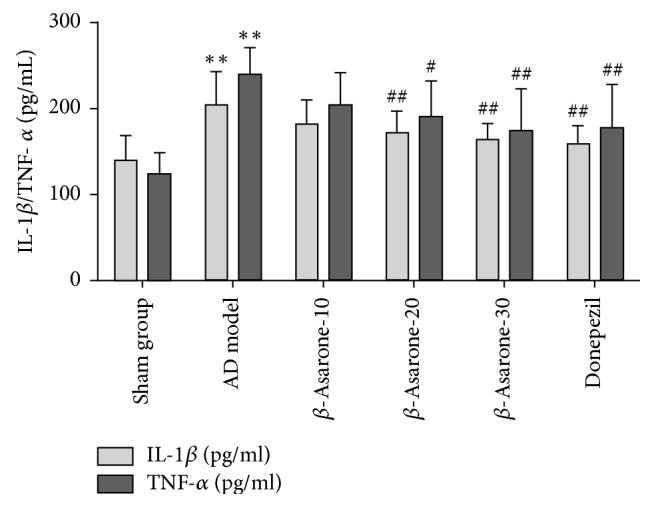
Effects of *β*-asarone on IL-1*β* and TNF-*α* levels in hippocampi of AD model rats induced by A*β*1–42.* Note*. Compared with the sham group, ^*∗∗*^*P* < 0.01; compared with the model group, ^#^*P* < 0.05, ^##^*P* < 0.01.

As shown in Figure 7, IL-1*β* and TNF-*α* levels were significantly elevated in hippocampi of the AD model group compared to those of the sham group (203.5 ± 39.50 versus 140.0 ± 28.27 pg/mL, 239.8 ± 30.89 versus 124.6 ± 24.47 pg/mL, *P* < 0.01, *P* < 0.01). After *β*-asarone treatment for 28 days, all of the indicators above were reduced. The level of IL-1*β* was decreased in groups receiving *β*-asarone (20 mg/kg, 30 mg/kg) treatment compared to the AD model group (171.7 ± 25.10 versus 203.5 ± 39.50 pg/mL, 163.3 ± 18.80 versus 203.5 ± 39.50 pg/mL, *P* < 0.01, *P* < 0.01). The level of TNF-*α* was decreased in groups receiving *β*-asarone (20 mg/kg, 30 mg/kg) treatment compared to the AD model group (189.8 ± 42.12 versus 239.8 ± 30.89 pg/Ml, 174.0 ± 48.28 versus 239.8 ± 30.89 pg/mL, *P* < 0.05, *P* < 0.01). These results indicated that hippocampal A*β*1–42 injections could induce brain inflammation and *β*-asarone could protect brains from this injury.

### 3.6. *β*-Asarone Alleviates Astrocyte Damage by Suppressing IL-1*β*, TNF-*α*, and AQP Expression

**Figure 8:**
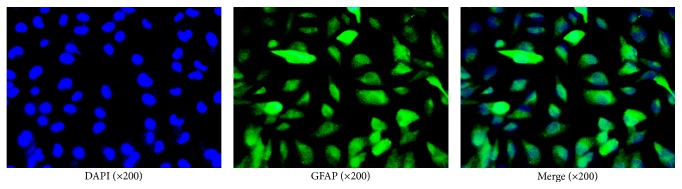
Immunofluorescence of GFAP to indicate that our cultured cells were astrocytes.

The immunofluorescence technique utilizing GFAP, an astrocyte marker, was used to determine that our cultured cells were astrocytes. As shown in Figure 8, GFAP-positive cells emitted green fluorescence, while nuclei emitted blue fluorescence. More than 95% of the cells were GFAP-positive, indicating that the cultured cells were indeed astrocytes.

**Figure 9:**
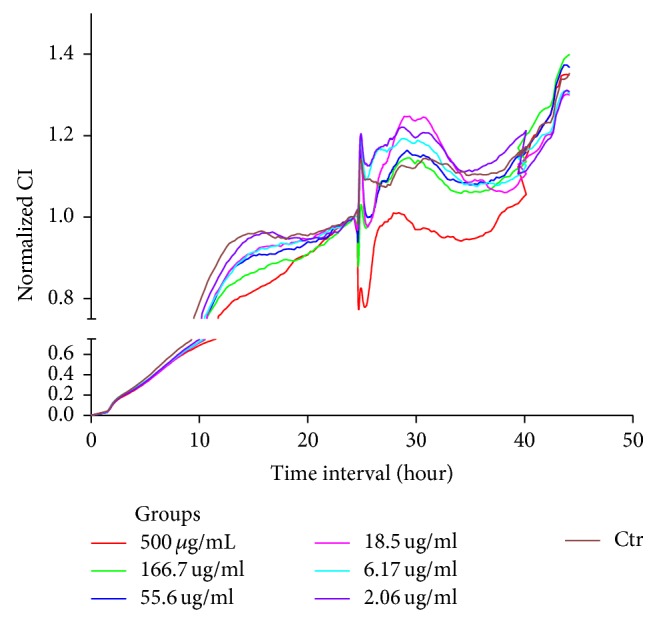
Effects of different concentrations of *β*-asarone on astrocyte cell viability.

**Figure 10:**
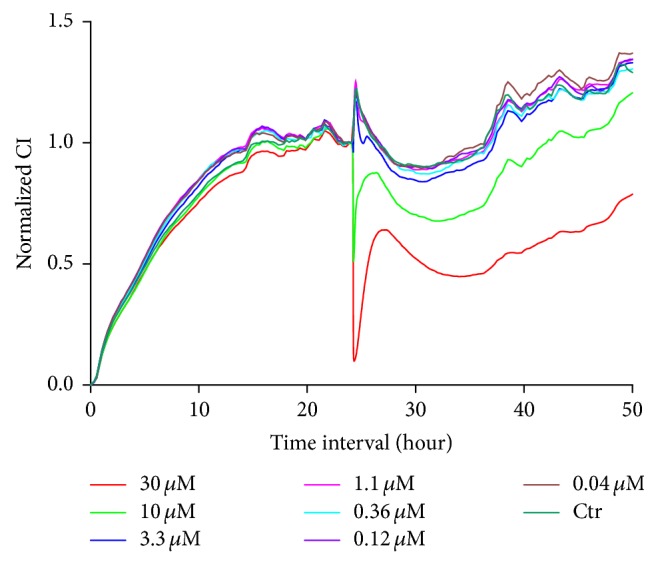
Effects of different concentrations of A*β*1–42 on astrocyte cell viability.

**Figure 11:**
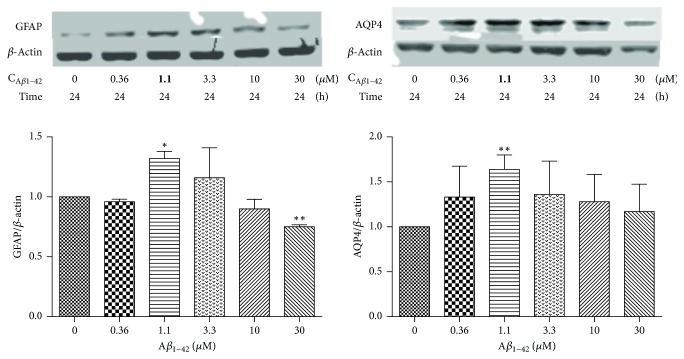
Effects of different concentrations of A*β*1–42 on the protein expression of AQP4 and GFAP in astrocytes.* Note*. Compared with the normal group, ^*∗*^*P* < 0.05, ^*∗∗*^*P* < 0.01.

**Figure 12:**
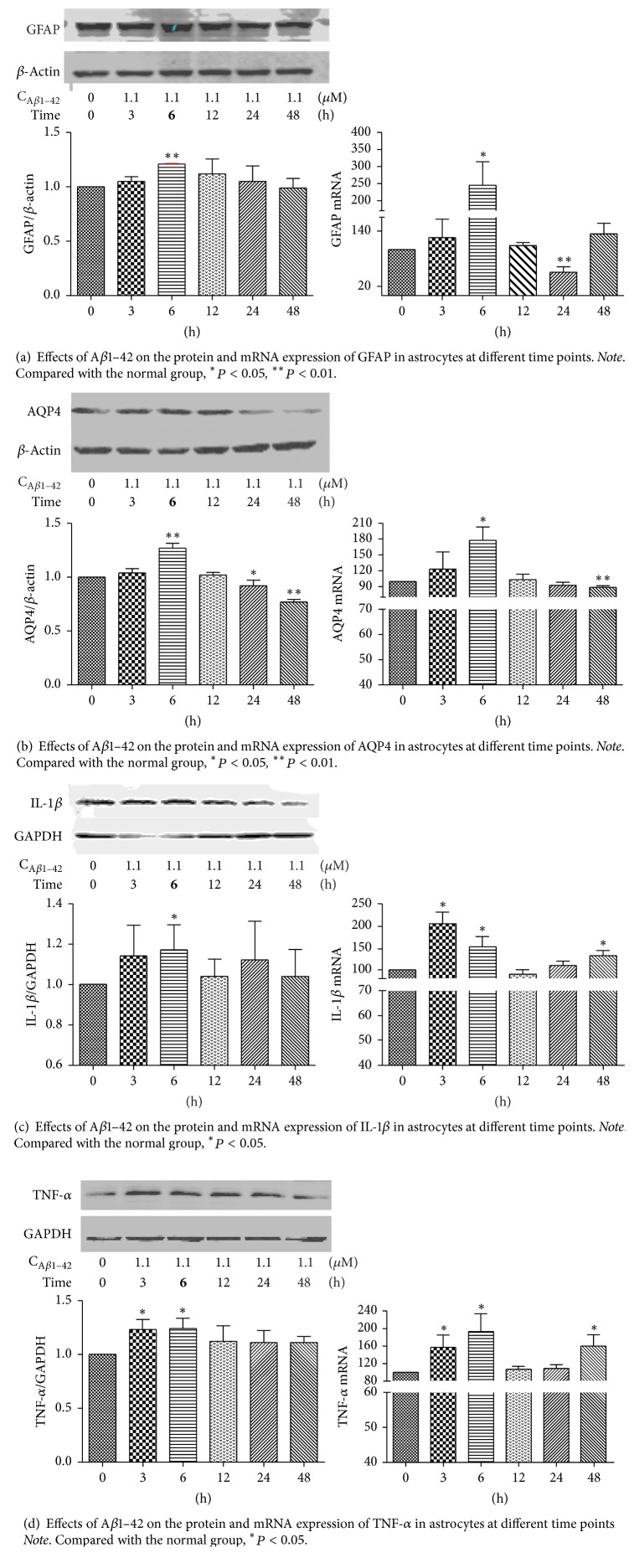


**Figure 13:**
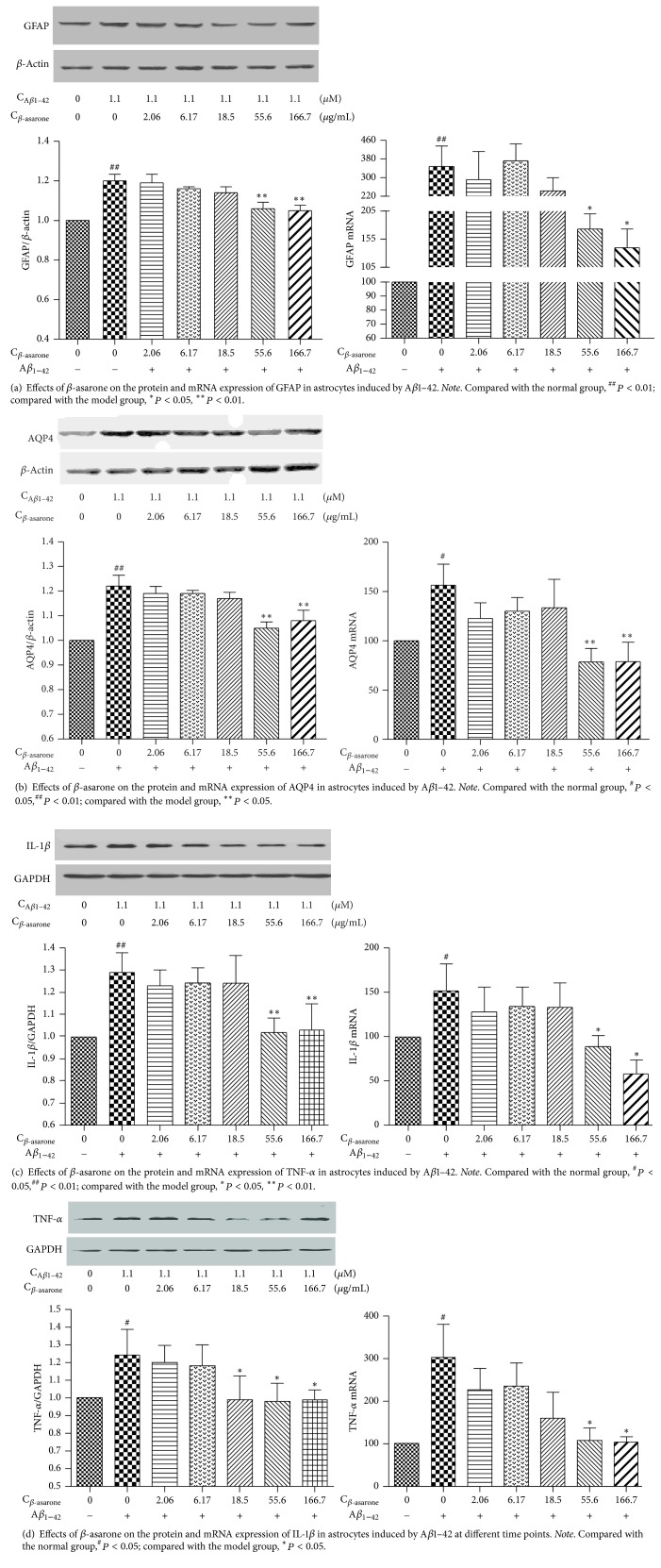


To evaluate the different concentrations of *β*-asarone and A*β*1–42 in RA-h cells, we performed a cell viability assay using RTCA. As shown in Figure 9, *β*-asarone treatment at 2.06, 6.17, 18.5, 55.6, and 166.7 *μ*g/mL had no effect on cell viability, while *β*-asarone treatment at 500 *μ*g/mL could partially inhibit astrocyte cell viability. Therefore, we adopted the 2.06, 6.17, 18.5, 55.6, and 166.7 *μ*g/mL *β*-asarone concentrations for use in subsequent studies. As shown in Figure 10, A*β*1–42 at 10 *μ*M and 30 *μ*M could significantly inhibit the cell viability. Furthermore, we used western blotting to observe the effects of A*β*1–42 on the protein expression of GFAP and AQP4. As shown in Figure 11, 1.1 *μ*M A*β*1–42 could significantly upregulate GFAP and AQP4 expression, and this A*β*1–42 concentration was thus chosen as the best stimulus concentration for subsequent studies. As shown in Figure 12, the protein expression levels of GFAP, AQP4, IL-1*β*, and TNF-*α* were significantly upregulated after 1.1 *μ*M A*β*1–42 treatment for 6 hours compared with those of the AD model group, and these results were similar to those of the mRNA expression analysis. Finally, as shown in Figure 13, we observed a protective effect of *β*-asarone on astrocytes treated with 1.1 *μ*M A*β*1–42 for 6 h. Compared with those of the AD model group, the protein expression levels of GFAP, AQP4, IL-1*β*, and TNF-*α* were significantly downregulated in the *β*-asarone treatment group (55.6, 166.7 *μ*g/mL), and these results were similar to those of the mRNA expression analysis. Together, these results indicate that *β*-asarone can protect astrocytes, most likely by inhibiting the expression of IL-1*β*, TNF-*α*, and AQP4.

## 4. Discussion

Alzheimer's disease is a neurodegenerative disorder and the most common form of dementia characterized by cognitive and memory impairment. During the past few decades, many plausible targets to treat AD, such as increasing choline uptake in the central nervous system, releasing acetylcholine from the hippocampus, increasing the activity or expression of choline acetyltransferase, and reducing the level of A*β* by regulating the balance between production and elimination, have been suggested. However, AD remains incurable due to its complex and multifactorial nature and lack of effective therapeutics. Therefore, continuing to look for new drugs to treat AD remains very necessary.

Although debates regarding the A*β* protein continue, new lines of evidence from laboratories and clinics worldwide support the concept that imbalance between the production and clearance of A*β*42 and related A*β* peptides is a very early, and often initiating, factor in AD [41]. In this experiment, we choose to use A*β*1–42 to build rat and cellular AD models.

Increasing evidence supports that astrocytes play important roles in AD [15–18, 42], suggesting that these cells regulate synaptic formation [43], transmission, and plasticity [44]. One study showed that astrocyte- and oligodendrocyte-specific genes, but not neuron-specific genes, shifted their regional expression patterns upon aging, particularly in the hippocampus and substantia nigra, while the expression of microglia- and endothelial-specific genes was increased in all brain regions analyzed in 480 individuals ranging in age from 16 to 106 years [45]. Other studies showed that aging causes morphological alterations in astrocytes and microglia [46]. In this study, we investigated the neuroprotective effects and mechanism of *β*-asarone against A*β*1–42-induced injury in astrocytes in vivo and in vitro.

HE and Nissl staining showed that CA1 region neurons in hippocampi of the model group were swollen, loose, and deeply stained, with pyknotic nuclei and vacuole-like structures being observed, while Nissl bodies were disorganized, swollen, and deformed, with condensed and deeply stained nuclei being observed. Congo red staining showed many A*β* deposits in hippocampi of the model rats. All the behavioral and pathological changes described above showed that the experimental model was successful, and were alleviated after treatment with *β*-asarone for four weeks. These results indicated that *β*-asarone could increase the cognitive ability and relieve AD pathological changes in rats.

Astrocytes are activated in response to acute or chronic central nervous system injury, and GFAP, a marker of astrocyte reactivity, is mainly expressed in activated astrocytes under pathological conditions [47]. Postmortem analyses of astrocytosis in human AD brain tissues have reported activated GFAP-positive astrocytes next to fibrillar A*β* plaques [15, 16], and one study found that GFAP was significantly age-related, as both the protein and mRNA levels of GFAP were increased in the hippocampus of aged senescence-accelerated-prone mice (SAMP8) and senescence-accelerated-resistant mice (SAMR1) [48]. In our study, we also observed GFAP-positive astrocytes to be significantly more abundant in hippocampi of the model rats. In addition, the number of GFAP-positive astrocytes was also dramatically increased after treatment with A*β*1–42. Meanwhile, *β*-asarone could reduce the expression of GFAP-positive astrocytes, indicating that *β*-asarone could reduce astrocyte reactivity.

Recent evidence also suggests that various A*β* complexes interact with microglial and astrocytic expression pattern recognition receptors, playing roles in innate immunity [49]. A*β* treatment of astrocyte-enriched cultures has a significant overall effect on the release of inflammatory cytokines [50], and activated astrocytes may increase A*β* generation [51]. In our experiment, with A*β*1–42 stimulation, TNF-*α* and IL-1*β* expression was increased significantly in both rats and astrocytes. In addition, *β*-asarone could reduce the levels of TNF-*α* and IL-1*β* in the brain tissues of AD rats. In addition, the gene and protein expression levels of TNF-*α* and IL-1*β* in astrocytes were reversed after treatment with *β*-asarone.

AQP4 is abundantly expressed in astrocytic vascular endfeet and plays important roles in regulating the physiological functions of astrocytes [24, 52], and AQP4 deficiency exacerbates brain oxidative stress [53] and memory deficits [26, 53]. Studies also showed that AQP4 plays a role in synaptic plasticity and that AQP4 deficiency impairs synaptic plasticity and spatial memory [54–56]. In addition, another study showed that lower concentrations of A*β*1–42 (0.1–1 *μ*M) increased AQP4 expression in cultured mouse cortical astrocytes, while higher concentrations of A*β*1–42 (10 *μ*M) decreased AQP4 expression. Furthermore, knocking out the AQP4 gene reduced A*β*1–42-induced astrocyte activation and apoptosis [28]. Moreover, another study showed that AQP4 was upregulated by proinflammatory cytokines in astrocytes [57]. Therefore, the regulation of astrocyte function by AQP4 may be a promising therapeutic strategy for AD. In this experiment, we observed the protein expression of AQP4 to be dramatically increased in the model rats, while the gene and protein expression levels of AQP4 in astrocytes were also dramatically increased after treatment with A*β*1–42 (1.1 *μ*M). In addition, these changes were partially reversed by treatment with *β*-asarone.

The results described herein show that *β*-asarone could alleviate the symptoms of AD by protecting astrocytes, and the underlying neuroprotective mechanism may be that *β*-asarone alleviates the activation of astrocytes by reducing the levels of TNF-*α* and IL-1*β* and then downregulating AQP4 expression. However, the mechanism underlying AQP4 regulation in astrocytes must be studied further, as it is undoubtedly a meaningful and challenging event.

## 5. Conclusions

The present study revealed that *β*-asarone could alleviate the symptoms of AD by protecting astrocytes, possibly by inhibiting TNF-*α* and IL-1*β* secretion and then downregulating AQP4 expression.

## Figures and Tables

**Table 1:** Primer sequences for RT-qPCR.

Gene	Forward primer	Reverse primer
GFAP	CTGAAACAGGAGAGAGGGACTT	TGAGCAACCAGGAATAGACCT
AQP4	CTCAGTGGGAAATGTAGCCTT	CGACCCTAACCAAGTCTCCT
IL-1*β*	GCCAACAAGTGGTATTCTCCAT	GTGCCGTCTTTCATCACACA
TNF-*α*	GCCGATTTGCCATTTCATAC	TGGAAGACTCCTCCCAGGTA
